# Dental caries prevalence in all school children in one of the grassroots areas in Southern China: a retrospective study

**DOI:** 10.3389/froh.2026.1774964

**Published:** 2026-03-04

**Authors:** Jin Sun, Zhiqiang Zhang, Yuting Liu, Liudan Peng, Huanhua Zhu, Zemo Gan

**Affiliations:** 1 Medical Affairs Department, The Affiliated Shenzhen Stomatology Hospital of Shenzhen University, Shenzhen, China; 2 Department of Stomatology, Zijin County Feng'an Health Center, Heyuan, China

**Keywords:** dental caries, epidemiology, oral health, pediatric dentistry, preventive dentistry

## Abstract

**Background:**

The prevalence of dental caries among minors has always been a major issue globally, especially in economically underdeveloped regions. To investigate the prevalence of dental caries among all school children in Feng'an Town, which is one of the typical grassroots areas in southern China, providing reference basis for the development of oral health care for children in grassroots areas.

**Materials and methods:**

Data of a total of 2,834 children from all kindergartens, primary schools, and middle schools in Feng'an Town, one of the grassroots area in southern China was surveyed in this retrospective study. The prevalence of dental caries and the mean Decayed, Missing, and Filled Teeth (DMFT/dmft) scores (an index used to measure the severity of dental caries) for deciduous and permanent teeth were statistically analyzed and reported. A Chi-square test was used to assess the differences between the caries-free and have-caries group. The Mann–Whitney *U*-test or Kruskal–Wallis *H*-test was used to evaluate mean DMFT/dmft score. The Benjamini-Hochberg procedure was used to correct the *P* values for False Discovery Rate (FDR). The Spearman's rank correlation coefficient (rs) was used to assess the trend relationship between ordinal variables (age, grade) and caries severity (DMFT/dmft score).

**Results:**

In kindergartens, 79% of the 481 children had deciduous teeth caries, with a mean dmft score of 6.69 ± 5.31, and age was a significant factor influencing caries prevalence and mean dmft score (*P* < 0.05). Primary schools showed a high combined prevalence of deciduous and permanent teeth caries at 88.5% among 1,504 children, with a combined mean DMFT/dmft score of 5.63 ± 4.15. Additionally, 42.6% of these children had permanent teeth caries, with a mean DMFT score of 1.04 ± 1.19. School, part of sex, and grade were significant factors affecting the mean DMFT/dmft and DMFT scores (*P* < 0.05). In middle schools, 75.9% of the 849 children had permanent teeth caries, with a mean DMFT score of 3.86 ± 3.85, and sex was a significant factor influencing caries prevalence and mean DMFT score (*P* < 0.05).

**Conclusion:**

The prevalence of dental caries among school children in grassroots areas in southern China is severe, necessitating attention and collaborative efforts from multiple sectors. Based on our findings, several policy measures could be considered to improve the oral health of children in grassroots areas. Firstly, monthly structured parental workshops in kindergartens led by certified dental hygienists. Secondly, implement school—based fluoride programs and atraumatic restorative treatments (ART). Thirdly, town government subsidize dental kits for low—income families. Fourthly, schools integrate daily supervised toothbrushing into schedules. Collaboratively, Guangdong Provincial Education Department allocate funds for annual dental check—ups and sealant programs. Finally, schools and health centers design flexible schedules with "dental slots" for emergency and preventive care.

## 1 Introduction

Dental caries is a chronic infectious disease that is widely prevalent worldwide, with a particularly high incidence rate among children and adolescents, and has become a significant public health issue affecting the oral health and overall development of this population group (1). Epidemiological studies have shown that dental caries not only directly leads to dental defects and pain but may also cause physiological impairments such as reduced masticatory function and nutritional intake disorders (2), thereby resulting in psychological and behavioral problems like decreased learning concentration and social interaction obstacles in learning and social interaction. These issues can have profound and far-reaching impacts on the academic performance of children and adolescents and the labor quality of adults (3). Although the World Health Organization has listed dental caries as one of the three major non-communicable diseases requiring prioritized prevention and control (4), due to factors such as uneven regional economic development levels and oral health resource allocation, the prevalence of dental caries among children and adolescents in rural areas of China remains high, and the prevention and control situation is severe (5).

Existing research has demonstrated significant regional disparities in the epidemiological characteristics of dental caries, with its distribution pattern closely related to regional social indicators, residents' health literacy, and the accessibility of medical resources (6). In contrast to the overall economic prosperity in southern China, where the economy is relatively well-developed, especially with Guangdong Province taking the lead, there are still pockets of underdeveloped areas. Feng'an Town, Zijin County, as a typical water source protection-oriented township in the eastern Guangdong region of southern China, is subject to restrictions imposed by ecological protection policies. The local economy is primarily based on traditional agriculture (7, 8), with a gross domestic product (GDP) less than 0.12% of that of Guangdong Province and residents' disposable income less than 0.33% of that of the province (9, 10). Local risk factors such as limited access to dental care facilities due to economic constraints and the possible high consumption of locally—produced sugary agricultural products may further contribute to this situation. Additionally, in southern China, the price of sugary snacks is relatively low, making them easily accessible to children in Feng'an Town. This unique socioeconomic background may shape a dental caries prevalence pattern with regional characteristics through influencing dietary patterns, oral hygiene habits, or medical behaviors. However, there is still a lack of epidemiological research on dental caries among children and adolescents in such ecologically protected and economically underdeveloped townships, and the absence of relevant data restricts the precise formulation of regional prevention and control strategies.

This study is a retrospective study surveying oral health data of all school children (including kindergarten to junior high school) in Feng'an Town, Zijin County. It comprehensively evaluated the dental caries status of deciduous and permanent teeth among children and adolescents in the area, and also compared it with Zijin County, Heyuan City, Guangdong Province, or national benchmarks. This research design combines timeliness and representativeness, enabling the acquisition of regional baseline data within a relatively short period and thus providing empirical evidence for revealing the prevalence patterns of dental caries among children and adolescents in grassroots areas. The research findings will contribute to constructing an oral health management model that aligns with the actual conditions of grassroots areas and provide scientific support for formulating differentiated dental caries prevention and control policies and optimizing the allocation of primary oral health resources.

## 2 Materials and methods

### 2.1 Data background

This is a retrospective study using data from the Department of Stomatology at Feng'an Health Center in Zijin County from 2024 to 2025. The data was from a dental examination conducted by dental personnel on school children [kindergarten, primary school, and Middle School (junior high only)] from all 10 schools in Feng'an Town, Zijin County. Zijin County Feng'an Health Center approved the use of its database and data collection in August 2025.

The data were organized and stored in a well-designed Excel spreadsheet. This process follows the requirements of the Office of the Dean of Feng'an Health Center in Zijin County, and is supervised by a dedicated team to ensure the confidentiality, integrity, and quality of the data.

The data supporting the results of this study can be obtained from Feng'an Health Center in Zijin County, but the availability of these data is limited. These data were used under the permission of this study and are therefore not publicly available. However, with reasonable requests and permission from Zijin County Feng'an Health Center, the author can provide data.

### 2.2 Study participants

Based on the fact that the database contains all children in all schools, the study participants were these all children within Feng'an Town, Zijin County, including 2 kindergartens (Feng'an Central Kindergarten and Xiashi Kindergarten), 7 primary schools (Feng'an Central Primary School, Xiashi Primary School, Shangkeng Primary School, Juemin Primary School, Foling Primary School, Huangdong Primary School, and Huanglong Primary School), and 1 middle school [Feng'an Middle School (junior high only)], with a total of 2,834 school children, comprising 481, 1,504 and 849 children respectively. This report follows to the STROBE (Strengthening the Reporting of Observational Studies in Epidemiology) statement guidelines for reporting cross-sectional studies.

### 2.3 Examiners

According to the records in the database of Zijin County Feng'an Health Center, there were four examiners responsible for this survey, all of whom were oral clinical practitioners. Prior to the implementation of the dental examination, the examiners underwent rigorous training with reference to the dental caries examination criteria from the Fourth National Oral Health Epidemiological Survey (5). All examiners passed the intra-examiner and inter-examiner reliability tests for dental caries examination standards, with Kappa values all exceeding 0.85.

### 2.4 Examination criteria

According to the records in the database of Zijin County Feng'an Health Center, all school children were examined in accordance with the dental caries examination criteria from the Fourth National Oral Health Epidemiological Survey (5). The examiner performs visual examination combined with probing under artificial light source. The environment is spacious and well lit. The tools include dental mirrors, cotton swabs and probes. The examination sequence is based on the four quadrants of the oral cavity.

### 2.5 Statistical analysis

Examiners utilized Feng'an Health Center designated work computer and WPS (version 12.1.0.18608, Kingsoft Office Software Corporation, China) to input the data. Subsequently, two field assistants conducted a thorough review of the data to prevent any input errors. Frequency and percentage [*N*(%)] were presented for dental caries prevalence, while Mean ± standard deviation (*N* ± SD) were presented for mean DMFT/dmft score. Data were analysed using SPSS version 26.0 for Windows (IBM Inc, Chicago, IL, USA). A Chi-square test was used to assess the differences between caries-free and have-caries group. Regarding the statistical analysis of the mean DMFT/dmft scores, we have conducted a preliminary assessment of the data and found that none of them followed a normal distribution. Therefore, within the kindergarten cohort, we employed the Mann–Whitney *U*-test for statistical analysis of school and sex factors, while utilizing the Kruskal–Wallis *H*-test for the analysis of age factors. Within the primary school cohort, the Mann–Whitney *U*-test was applied for the statistical analysis of sex factors, and the Kruskal–Wallis *H*-test was used for the analysis of school and grade factors. Within the middle school cohort, the Mann–Whitney *U*-test was employed for the statistical analysis of sex factors, and the Kruskal–Wallis H-test was utilized for the analysis of grade factors. A 95% confidence interval (95% CI) and effect sizes were calculated, and the significance level was set at *p* < 0.05.

To control the risk of type I error (false positive) that may arise from multiple statistical tests, we employed the Benjamini-Hochberg method to correct the *P* values for False Discovery Rate (FDR). All significant results reported in this study were determined based on the corrected *P* values (Adj. P).

We calculated the 95% confidence interval (95% CI) and effect size to assess the practical significance of the difference. Specifically, for the comparison of categorical variables (caries prevalence), Cramer's V was used to measure the effect size; for quantitative data with non-normal distribution (DMFT/dmft scores), Rank-biserial correlation (r) (for comparison between two groups) or Epsilon-squared (ɛ^2^) (for comparison among multiple groups) was calculated.

To assess the trend relationship between ordinal variables (age, grade) and caries severity (DMFT/dmft score), we calculated the Spearman's rank correlation coefficient (rs).

## 3 Results

### 3.1 Dental caries status among kindergarten children

**Figure 1:**
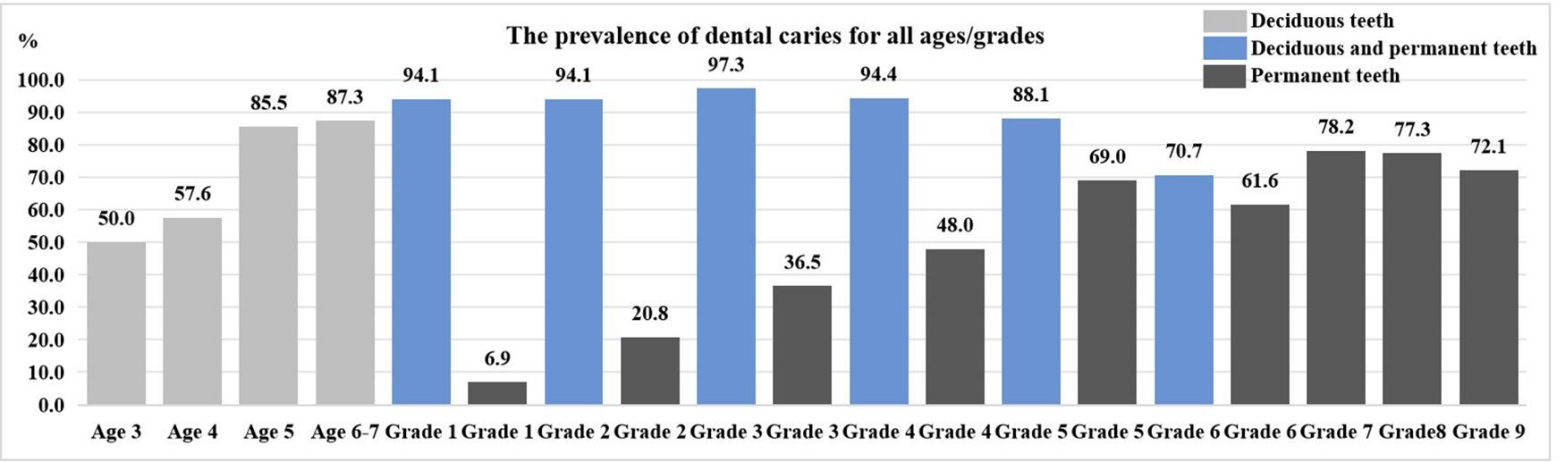
The prevalence of dental caries for all ages/grades (%).

**Figure 2:**
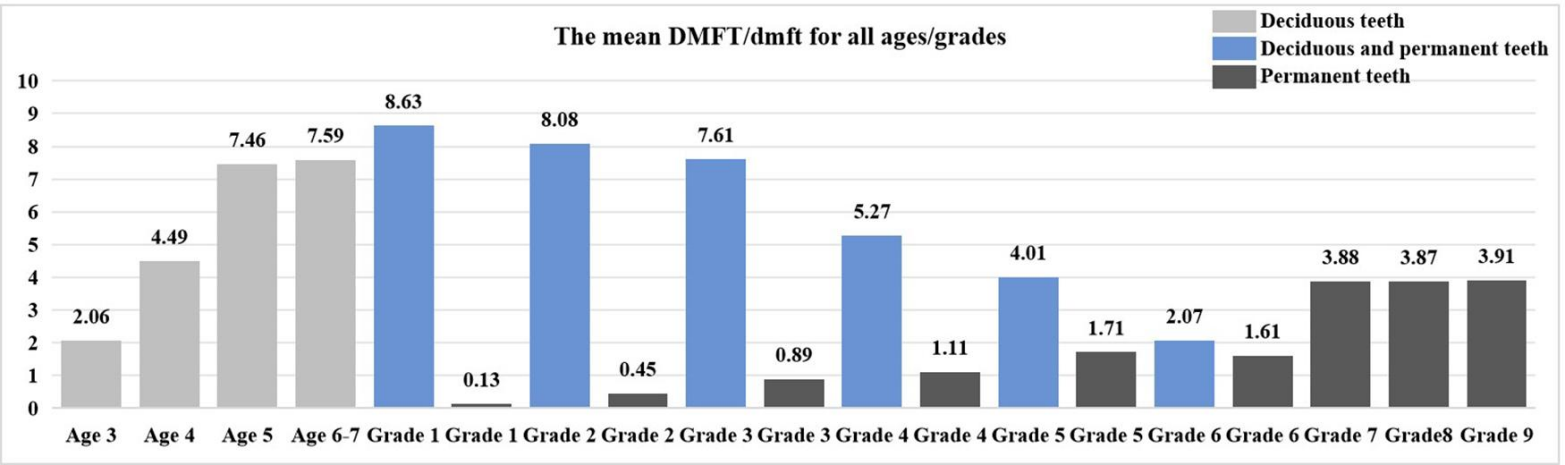
The mean DMFT/dmft for all ages/grades.

A total of 2 local kindergartens were examined, with 481 participants, and the number of males and females was roughly equal. The prevalence of dental caries in deciduous teeth was 79%, the mean dmft score for deciduous teeth was 6.69 ± 5.31, and the Significant Caries Index (SiC) was 12.51. Among them, the prevalence of dental caries in deciduous teeth among the age 3 was 50%, with a mean dmft score of 2.06 ± 2.8; the prevalence among the age 5 was 85.5%, with a mean dmft score of 7.46 ± 5.45. Within the age range of 3–7 years, both the prevalence and the mean dmft score of dental caries in deciduous teeth showed an increasing trend with age (the mean dmft score rs = 0.250, *P* < 0.001). And the differences have statistical significance (*p* < 0.05) (Table 1, Figures 1, 2).

**Table 1:** Deciduous teeth caries status in kindergartens.

Kindergartens (*n*)	Caries-free *N*	Have caries *N* (%)	*p*-value (Adj. *P*)	CI	Effect size	Mean Dmft (±SD)	*p*-value (Adj. *P*)	CI	Effect size
Total(481)	101	380 (79.0)				6.69 ± 5.31			
Schools			0.062	−0.16, 0.006	0.085		0.062	−0.047, 1.967	0.18
			(0.093)				(0.093)		
Central Kindergarten(337)	63	274 (81.3)				6.98 ± 5.31			
Xiashi Kindergarten(144)	38	106 (73.6)				6.02 ± 5.28			
Sexes			0.141	−0.017, 0.127	0.067		0.938	−2.47, 2.21	0.03
			(0.169)				(0.938)		
Male(240)	57	183 (76.2)				6.76 ± 5.58			
Female(241)	44	197 (81.7)				6.62 ± 5.04			
Ages			<0.001	−0.076, 0.518	0.192		<0.001	−8.26, 1.07	0.11
			(0.003)				(0.003)		
3 (18)	9	9 (50.0)				2.06 ± 2.8			
4 (99)	42	57 (57.6)				4.49 ± 4.99			
5 (207)	30	177 (85.5)				7.46 ± 5.45			
6–7 (157)	20	137 (87.3)				7.59 ± 4.9			

(*N*, *n*, population size; SD, standard deviation; CI, confidence interval. Adj. *P*: *Adjustment p*-value. Caries prevalence: Using the chi-square test, the reported effect size is Cramer's V; Mean dmft: For comparisons between two groups (school, sex): Using the Mann–Whitney *U* test, the reported effect size is the rank-biserial correlation (r); For comparisons among multiple groups (age): Using the Kruskal–Wallis *H* test, the reported effect size is Epsilon-squared (ɛ²).

### 3.2 Dental caries status among primary school children

A total of 7 local primary schools were examined, with 1,504 participants, including 789 male children and 715 female children. The prevalence of dental caries in deciduous and permanent teeth was 88.5%, with a mean DMFT/dmft score of 5.63 ± 4.15, and the SiC was 10.63. The prevalence of dental caries in permanent teeth was 42.6%, with a mean DMFT score of 1.04 ± 1.19, and the SiC was 6.60. Shangkeng Primary School had the highest prevalence of dental caries in both deciduous and permanent teeth (94.3%) and mean DMFT/dmft score (6.29 ± 3.87), while Huanglong Primary School had the highest prevalence of dental caries in permanent teeth (56.7%) and mean DMFT score (1.48 ± 1.66). As the grade level increased, the prevalence of dental caries in both deciduous and permanent teeth and mean DMFT/dmft score both showed an approximate downward trend (the mean DMFT/dmft score rs = −0.588, *P* < 0.001). The prevalence of dental caries in permanent teeth and mean DMFT score showed an overall increasing trend (the mean DMFT score rs = 0.407, *P* < 0.001). Notably, school, part of sex and grade demonstrates statistically significant differences in the dental caries prevalence, mean DMFT/dmft and DMFT score (*p* < 0.05) (Tables 2, 3, Figures 1, 2).

**Table 2:** Deciduous and permanent teeth caries status in primary schools.

Primary schools (*n*)	Caries-free *N*	Have caries *N* (%)	*p*-value (Adj. *P*)	CI	Effect size	Mean DMFT/dmft (±SD)	*p*-value (Adj. *P*)	CI	Effect size
Total(1,504)	173	1,331 (88.5)				5.63 ± 4.15			
Schools			<0.001	−0.048, 0.133	0.064		<0.001	−0.145, 2.84	0.03
			(0.002)				(0.002)		
Central Primary School(699)	94	605 (86.5)				5.48 ± 4.23			
Xiashi Primary School(287)	37	250 (87.1)				5.61 ± 4.08			
Shangkeng Primary School(177)	10	167 (94.3)				6.29 ± 3.87			
Juemin Primary School(131)	9	122 (93.1)				6.21 ± 4.57			
Foling Primary School(75)	13	62 (82.7)				3.87 ± 3.23			
Huangdong Primary School(75)	6	69 (92.0)				6.08 ± 4.47			
Huanglong Primary School(60)	4	56 (93.3)				5.97 ± 3.40			
Sexes			0.047	−0.004, 0.058	0.051		0.794	−0.27, 0.35	0.01
			(0.056)				(0.794)		
Male(789)	101	688 (87.2)				5.61 ± 4.16			
Female(715)	72	643 (89.9)				5.65 ± 4.15			
Grades			<0.001	−0.004, 0.058	0.05		<0.001	−0.41, 7.5	0.43
			(0.002)				(0.002)		
1 (219)	13	206 (94.1)				8.63 ± 4.50			
2 (236)	14	222 (94.1)				8.08 ± 3.83			
3 (219)	6	213 (97.3)				7.61 ± 3.46			
4 (250)	14	236 (94.4)				5.27 ± 3.22			
5 (252)	30	222 (88.1)				4.01 ± 3.06			
6 (328)	96	232 (70.7)				2.07 ± 2.10			

(*N*, *n*, population size; SD, standard deviation; CI, confidence interval. Adj. *P*: *Adjustment p*-value. Prevalence of dental caries: Chi-square test was used, and the reported effect size was Cramer's V; mean DMFT/dmft: For comparisons between two groups (sex): Using the Mann–Whitney *U* test, the reported effect size is the rank-biserial correlation (r); for comparisons among multiple groups (schools, grades): Using the Kruskal–Wallis *H* test, the reported effect size is Epsilon-squared (ɛ²).

**Table 3:** Permanent teeth caries status in primary schools.

Primary schools (*n*)	Caries-free *N*	Have caries *N* (%)	*p*-value (Adj. *P*)	CI	Effect size	Mean DMFT (±SD)	*p*-value (Adj. *P*)	CI	Effect size
Total(1,504)	864	640 (42.6)				1.04 ± 1.19			
Schools			<0.001	0.298, 0.694	0.30		<0.001	−1.32, 0.48	0.09
			(0.001)				(0.001)		
Central Primary School(699)	462	237 (33.9)				0.71 ± 1.20			
Xiashi Primary School(287)	153	134 (46.7)				1.21 ± 1.52			
Shangkeng Primary School(177)	93	84 (47.5)				1.29 ± 1.71			
Juemin Primary School(131)	57	74 (56.5)				1.76 ± 1.94			
Foling Primary School(75)	35	40 (53.3)				1.08 ± 1.23			
Huangdong Primary School(75)	38	37 (49.3)				1.29 ± 1.65			
Huanglong Primary School(60)	26	34 (56.7)				1.48 ± 1.66			
Sexes			<0.001	0.335, 0.526	0.12		<0.001	0.21, 0.51	0.24
			(0.001)				(0.001)		
Male(789)	499	290 (36.8)				0.87 ± 1.37			
Female(715)	365	350 (48.9)				1.23 ± 1.58			
Grades			<0.001	0.039, 0.744	0.48		<0.001	−0.185, 0.57	0.27
			(0.001)				(0.001)		
1 (219)	204	15 (6.9)				0.13 ± 0.56			
2 (236)	187	49 (20.8)				0.45 ± 1.02			
3 (219)	139	80 (36.5)				0.89 ± 1.36			
4 (250)	130	120 (48)				1.11 ± 1.38			
5 (252)	78	174 (69)				1.71 ± 1.53			
6 (328)	126	202 (61.6)				1.61 ± 1.80			

(*N*, *n*, population size; SD, standard deviation; CI, confidence interval. Adj. *P*: *Adjustment p*-value. Prevalence of dental caries: Chi-square test was used, and the reported effect size was Cramer's V; mean DMFT: For comparisons between two groups (sex): Using the Mann–Whitney *U* test, the reported effect size is the rank-biserial correlation (r); for comparisons among multiple groups (schools, grades): Using the Kruskal–Wallis *H* test, the reported effect size is Epsilon-squared (ɛ²).

### 3.3 Dental caries status among middle school children

A total of 1 local middle school was examined, with 849 participants, including 451 male children and 398 female children. The prevalence of dental caries in permanent teeth was 75.9%, with mean DMFT score of 3.86 ± 3.85, and the SiC was 8.70. Among them, the prevalence of dental caries in permanent teeth among the 12-year-old group was 78.2%, with mean DMFT score of 3.88 ± 3.93. As the grade level increased, the prevalence of dental caries in permanent teeth decreased, while the mean DMFT score shows stability (the mean DMFT score rs = −0.004, *P* = 0.907) (Figures 1, 2). Among the population, female children had a significantly higher mean DMFT score (*p* < 0.05) (Table 4).

**Table 4:** Permanent teeth caries status in middle school.

Middle school (*n*)	Caries-free *N*	Have caries *N* (%)	*p*-value (Adj. *P*)	CI	Effect size	Mean DMFT (±SD)	*p*-value (Adj. *P*)	CI	Effect size
Total(849)	205	644 (75.9)				3.86 ± 3.85			
Sexes			<0.05 (0.1)	0.012, 0.13	0.15		<0.001 (0.004)	0.39, 1.37	0.20
Male(451)	124	327 (72.5)				3.47 ± 3.75			
Female(398)	81	317 (79.6)				4.35 ± 3.93			
Grades			0.25 (0.333)	−0.021, 0.125	0.11		0.955 (0.955)	−0.57, 0.54	0.0001
7 (280)	61	219 (78.2)				3.88 ± 3.93			
8 (282)	64	218 (77.3)				3.87 ± 3.7			
9 (287)	80	207 (72.1)				3.91 ± 3.94			

(*N*, *n*, population size; SD, standard deviation; CI, confidence interval. Adj. *P*: *Adjustment p*-value. Prevalence of dental caries: Chi-square test was used, and the reported effect size was Cramer's V; mean DMFT: For comparisons between two groups (sex): Using the Mann–Whitney *U* test, the reported effect size is the rank-biserial correlation (r); for comparisons among multiple groups (grades): Using the Kruskal–Wallis *H* test, the reported effect size is Epsilon-squared (ɛ²).

## 4 Discussion

Dental caries is a highly prevalent chronic disease among children and adolescents, negatively impacting their dental development, chewing function, nutritional intake, growth and development, and overall quality of life (1–3). This study aimed to comprehensively understand the dental caries status of children in Feng'an Town, Zijin County, one of the grassroots areas in southern China, through large-scale surveys and systematic statistical analysis, providing a scientific basis for developing targeted dental caries prevention strategies in the grassroots areas. The results revealed a severe prevalence of dental caries in both deciduous and permanent teeth among the examined children, highlighting the urgency of oral health issues among local children and adolescents.

The main findings indicated that among 481 children in kindergartens in the area, the prevalence of dental caries in deciduous teeth was 79%, with mean dmft score of 6.69 ± 5.31. It is worth noting that in order to compare with the reports of most scholars and The Fourth National Oral Health Epidemiological Survey Report, we focus on reporting the dental caries status of children aged 3 and 5. The prevalence among 3-year-olds was 50%, with a mean dmft score of 2.06 ± 2.8, while among 5-year-olds, it was 85.5%, with a mean dmft score of 7.46 ± 5.45. Compared to Zijin County (20.83%–68.94%) (11), Heyuan City (32%–79%) (12), Guangdong Province (58.33%–78.47%) (13), and the national 5-year-old prevalence (70.9%) (5), the prevalence of dental caries in deciduous teeth among kindergarten children in Feng'an Town, Zijin County, was higher, indicating a severe situation of dental caries in deciduous teeth among children at the kindergarten stage in the area. This suggests the high strategic importance of early intervention and prevention for young children in the region. The occurrence of dental caries in deciduous teeth is associated with multiple factors, particularly poor dietary habits and a lack of parental guidance in oral hygiene practices (14). Frequent consumption of high-sugar foods and beverages by kindergarten children and parents' failure to effectively guide their oral hygiene habits can contribute to the high prevalence of dental caries in deciduous teeth (15). Therefore, to address early childhood caries, a multi-pronged approach integrating parental education and non-invasive dental care is essential. First, structured parental workshops could be conducted monthly in kindergartens, led by certified dental hygienists. These sessions must focus on practical skills, such as demonstrating age-appropriate toothbrushing techniques using anatomical models and distributing illustrated guides on reducing sugar intake. Complementing these efforts, mobile dental units from Feng'an Health Center could visit kindergartens biannually to provide free fluoride varnish applications and atraumatic restorative treatments (ART) for cavities using glass ionomer cement. To ensure accessibility, the town government could subsidize dental kits containing fluoride toothpaste, child-sized toothbrushes, and two-minute timers for low-income families, thereby addressing resource disparities.

At the primary school stage, among 1,504 children from seven examined schools, the prevalence of dental caries in deciduous and permanent teeth was 88.5%, with mean DMFT/dmft score of 5.63 ± 4.15. The prevalence of dental caries in permanent teeth was 42.6%, with mean DMFT score of 1.04 ± 1.19. Compared to Heyuan City (34%–69%) (16, 17) and Guangdong Province (30.56%–31.94%) (18), the prevalence of dental caries in both deciduous and permanent teeth among primary school children in Feng'an Town, Zijin County, was significantly higher, further confirming the prevalence of oral health issues in the area. Therefore, improving oral health literacy and resource allocation among primary school children requires targeted school-based programs and policy advocacy. Schools should integrate daily supervised toothbrushing sessions into class schedules, utilizing designated “oral health corners” equipped with mirrors, timers, and educational posters. Collaboratively, the Guangdong Provincial Education Department's work team in Feng'an Town should allocate funds for annual dental check-ups by visiting dentists and subsidized sealant programs targeting first permanent molars. To reduce sugar consumption, a “Sweet Swap” initiative could be launched, partnering with local stores to promote healthier alternatives (e.g., fruit snacks instead of candy) through school newsletters and classroom discussions. These measures collectively create a supportive environment for sustaining oral hygiene habits.

At the middle school stage, among 849 children from the only examined school, the prevalence of dental caries in permanent teeth was 75.9%, with a mean DMFT score of 3.86 ± 3.85. It is worth noting that in order to compare with the reports of most scholars and The Fourth National Oral Health Epidemiological Survey Report, we also focus on reporting the dental caries situation of 12-year-old children. Among the 12-year-old group, the prevalence was 78.2%, with a mean DMFT score of 3.88 ± 3.93. Compared to Guangdong Province (28.33%–47.29%) (19) and the national 12-year-old prevalence of permanent teeth (34.5%) (5), the prevalence of dental caries in permanent teeth among middle school children in Feng'an Town, Zijin County, was significantly higher, indicating a more severe situation of dental caries in permanent teeth among children at the middle school stage in the area. This suggests an urgent need for effective treatment and prevention measures. Oral diseases not only affect adolescents' physical health but also have negative impacts on their psychological and social functioning. Dental pain and aesthetic issues caused by dental caries may lead to psychological problems such as low self-esteem and anxiety in adolescents, thereby affecting their social activities (20). Furthermore, balancing academic demands with oral health maintenance poses unique challenges for adolescents. School should collaborate with health centers to design flexible scheduling systems, offering “dental slots” during free periods or after school for emergency care (e.g., pain relief) and preventive services (e.g., scaling, fluoride treatments). Peer-led education programs can enhance engagement, where trained student ambassadors deliver short oral health presentations during homeroom classes. Additionally, integrating mental health support is critical; psychologists could address stress-related bruxism through mindfulness sessions, linking oral health to holistic well-being. These strategies ensure adolescents receive timely care without disrupting academic priorities.

After specific statistical analysis, in the primary and middle school population, it was found that there were part of the sex differences in the prevalence of dental caries in deciduous and permanent teeth or permanent teeth and mean DMFT score (*p* < 0.05), except for mean DMFT/dmft score (*p* = 0.794) (Tables 2–4). This result suggests that when developing oral health education and prevention strategies, sex factors need to be fully considered to provide personalized oral health guidance. Tailoring interventions to sex-based behavioral differences can optimize outcomes. For male children, gamification techniques provided by the Guangdong Provincial Education Department's work team in Feng'an Town—such as “Brushing Challenge” apps with reward systems for consistent use—may improve adherence to oral hygiene routines. Distributing masculine-themed dental kits (e.g., sports-themed toothbrush holders) could further incentivize participation. Conversely, female children may benefit from targeted guidance on managing sweet food cravings, such as providing discreet sugar-free gum and snack-swapping guides in cafeterias. These sex-sensitive approaches acknowledge cultural and behavioral nuances, enhancing intervention effectiveness.

The high prevalence of dental caries among children at all levels of schools suggests the presence of multiple risk factors, possibly involving socioeconomic and environmental aspects. Notably, due to economic reasons in grassroots areas, the main population structure in Feng'an Town, Zijin County, consists of the older adults and children, with most parents working outside the region (21), leading to a severe lack of oral health care and education for children. This special population structure and social environment make children's oral health issues more prominent. To improve this situation, health centers have attempted to conduct health education and free clinics in kindergartens and primary schools, but further enhancement in intensity and scope is needed with the assistance and leadership of the Guangdong Provincial Education Department's work team in Feng'an Town and the town government. Additionally, incorporating student dental caries into public health projects, using public health supervision methods to monitor student dental caries status, and reminding timely treatment and maintenance are also effective ways to improve the oral health level of children and adolescents in townships. Moreover, with the extensive application of artificial intelligence (AI) technology in the field of dentistry, people in grassroots areas are able to utilize oral health AI advisor systems for self-monitoring of their oral health. The specific concept is as follows (22): the Guangdong Provincial Education Department will take the lead in collaborating with provincial-level oral specialty hospitals to jointly develop a mobile application. Elderly users only need to use their mobile phones to take a few photos of children' teeth. The AI program embedded in the application can then instantly analyze these photos and generate a report on dental caries. Subsequently, this report will be uploaded to the provincial-level oral specialty hospital for further professional analysis. After the analysis is completed, the hospital will transmit personalized oral care methods and treatment recommendations back to the mobile phones of the elderly users. At the same time, the relevant data of the children will also be sent to the Feng'an Health Center in Zijin County, which will be responsible for arranging subsequent appointment treatments and oral health maintenance work.

In further analysis, This study further revealed the severe inequality in the burden of dental caries among children of different school age groups by calculating the SiC Index, providing a key basis for the allocation of public health resources. The SiC, as a supplementary indicator to the mean DMFT/dmft score, effectively quantifies the extreme differences in the distribution of dental caries within a population by focusing on the top 30% of individuals with the most severe dental caries. The results showed that the SiC of primary teeth in kindergarten children (12.51) was significantly higher than their mean dmft score (6.69), indicating that although the average level of dental caries was high, a small number of children bore a heavier disease burden (such as SiC/dmft ratio reaching 1.87), suggesting that early intervention should prioritize high-risk groups. Similarly, the ratio of SiC (10.63) to DMFT/dmft (5.63) for deciduous and permanent teeth in primary schools (1.89) further confirms the persistent inequality of dental caries during the mixed dentition stage; The ratio of SiC (6.60) to DMFT (1.04) in the primary school permanent teeth group (6.35), although higher due to the overall mild dental caries in the sample, still reflects extreme inequality even at low levels of dental caries. Although the SiC (8.70) of permanent teeth in middle school is lower than that in kindergartens and primary schools, its ratio to DMFT (3.86) (2.25) indicates that with age, although the inequality of dental caries is alleviated, the burden of dental caries in high-risk individuals cannot be ignored. From a public health perspective, the introduction of the SiC strengthens the necessity of “precision prevention”. For example, the SiC in kindergartens and primary schools are significantly higher than the mean DMFT score, indicating that traditional intervention strategies guided by group average levels may not be sufficient to reduce inequality. Risk assessment tools need to be used to identify high SiC contribution subgroups and implement targeted measures. In addition, the decrease in the SiC to DMFT ratio during middle school may reflect the cumulative effect of public health interventions, but the SiC of 8.70 still suggests the need to continue to pay attention to the impact of changes in oral health behavior during adolescence on high-risk individuals. Future research can further combine social determinants to provide more detailed evidence for formulating policies to eliminate inequality in dental caries.

The strength of this study lies in its high universality and accuracy, as the survey objects cover all children in school across the town, comprehensively reflecting the dental caries status of children and adolescents in Feng'an Town, Zijin County. However, this study exhibits notable limitations primarily stemming from the absence of comprehensive behavioral data, particularly regarding children' daily oral hygiene routines (e.g., frequency and duration of toothbrushing, use of fluoridated toothpaste, and interdental cleaning practices) and detailed dietary patterns (including frequency of sugar-rich snack consumption, timing of carbohydrate intake relative to oral hygiene, and beverage choices). Furthermore, due to the lack of detailed data on key covariates such as oral hygiene behaviors and dietary habits, this study did not construct a multifactor regression model for risk factor analysis. A model that only includes demographic characteristics and ignores behavioral factors may not adequately control for confounding factors, leading to statistical bias. Therefore, this study focuses on providing descriptive epidemiological characteristics that have undergone rigorous statistical correction. Given that these factors constitute key modifiable risk elements in dental caries pathogenesis, our future research will integrate structured questionnaires to systematically capture these variables, thereby enabling a more nuanced analysis of how hygiene behaviors and dietary habits interact with socioenvironmental factors to influence caries prevalence in township populations. In addition, although two field assistants were assigned during the data entry stage to thoroughly verify the data to prevent input errors, and the physical examiners and school staff verified the student list, there is an inevitable risk that the original records may be incomplete or there may be administrative omissions in retrospective studies of the database. What is more, in this database, only the locations of decayed teeth were recorded, without missing (due to cares) and filled teeth, resulting in a decrease in the comprehensiveness of the study. Therefore, in future examinations, we suggest adding specific records of DMFT. Of course, we also reflect that the occurrence of unrecorded cases (filled teeth) is largely due to local children or parents having extremely low willingness or conditions for dental treatment. Meanwhile, This study only evaluated the current status of dental caries in students through clinical examinations, but did not collect potential influencing factors such as social behavior, diet, and family environment through survey questionnaires. This limitation may limit the depth and breadth of the research conclusions. Due to the lack of such data, this study was unable to analyze the independent contributions of these factors to dental caries, and it is also difficult to rule out residual confounding bias (such as overestimating the association between age and dental caries without controlling for a high sugar diet). Future research needs to supplement structured questionnaires that include variables such as daily brushing frequency, intake of sugary drinks, and household income, and further explore the interaction between individual and group level factors through a multi-level model to more accurately identify intervention targets for dental caries.

## 5 Conclusions

This study indicates a generally high prevalence of dental caries among children and adolescents in Feng'an Town, Zijin County, one of the grassroots areas in southern China, emphasizing the necessity of oral health education and prevention. In the future, it is essential to further develop appropriate technologies and policy measures suitable for grassroots areas to effectively improve the oral health level of local children and adolescents.

## Data Availability

The original contributions presented in the study are included in the article/Supplementary Material, further inquiries can be directed to the corresponding author.
