# Correction: Dental caries prevalence in all school children in one of the grassroots areas in southern China: a retrospective study

**DOI:** 10.3389/froh.2026.1824405

**Published:** 2026-03-24

**Authors:** Jin Sun, Zhiqiang Zhang, Yuting Liu, Liudan Peng, Huanhua Zhu, Zemo Gan

**Affiliations:** 1 Medical Affairs Department, The Affiliated Shenzhen Stomatology Hospital of Shenzhen University, Shenzhen, China; 2 Department of Stomatology, Zijin County Feng’an Health Center, Heyuan, China

**Keywords:** dental caries, epidemiology, oral health, pediatric dentistry, preventive dentistry

In the article, the funder “The General Program of Shenzhen Natural Science Foundation, JCYJ20250604190227035” to author Jin Sun was erroneously omitted.

The original version of this article has been updated.

In the Acknowledgements, the funder was omitted. The correct Acknowledgements is “Feng'an Town People's Government, all schools and Guangdong Provincial Education Department's work team in Feng'an Town. The Stomatology Project under the Integration of Medical and Preventive Services of Shenzhen Health Commission. This work was supported by The General Program of Shenzhen Natural Science Foundation [Grant No. JCYJ20250604190227035] for the project titled “Mechanistic Study on EphrinB2/EphB4 Synergizing with PDGF-BB/PDGFRβ to Regulate Dental Pulp Stem Cells for Promoting Angiogenesis.”

The original version of this article has been updated.

